# McArdle Disease and Exercise Physiology

**DOI:** 10.3390/biology3010157

**Published:** 2014-02-25

**Authors:** Yu Kitaoka

**Affiliations:** Department of Sports Sciences, The University of Tokyo, Komaba 3-8-1, Meguro-ku, Tokyo 153-8902, Japan; E-Mail: kitaoka@idaten.c.u-tokyo.ac.jp; Tel.: +81-3-5454-6858; Fax: +81-3-5454-4317

**Keywords:** McArdle disease, skeletal muscle, metabolism

## Abstract

McArdle disease (glycogen storage disease Type V; MD) is a metabolic myopathy caused by a deficiency in muscle glycogen phosphorylase. Since muscle glycogen is an important fuel for muscle during exercise, this inborn error of metabolism provides a model for understanding the role of glycogen in muscle function and the compensatory adaptations that occur in response to impaired glycogenolysis. Patients with MD have exercise intolerance with symptoms including premature fatigue, myalgia, and/or muscle cramps. Despite this, MD patients are able to perform prolonged exercise as a result of the “second wind” phenomenon, owing to the improved delivery of extra-muscular fuels during exercise. The present review will cover what this disease can teach us about exercise physiology, and particularly focuses on the compensatory pathways for energy delivery to muscle in the absence of glycogenolysis.

## 1. Introduction

McArdle disease (glycogen storage disease Type V; MD) is a myopathy caused by genetic defects in myophosphorylase, the skeletal muscle isoform of the enzyme glycogen phosphorylase. The first case was described in 1951 by Brian McArdle [1]. MD is now known as one of the most common disorders of muscle metabolism, with an estimated prevalence of approximately 1 per 100,000. The genetic defects that result in MD are autosomal recessive, and heterozygotes are usually asymptomatic. The myophosphorylase gene (*PYGM*) is on chromosome 11q13 [2,3], and more than 100 mutations have been detected according to the Human Gene Mutation Database [4]; this number is continually increasing as genetic technology advances. Currently, the p.R50X nonsense mutation (originally known as p.R49X) is the most frequently found mutation among Caucasian patients in North America [5] and Europe [6,7,8,9,10]. Other mutations are seen in specific ethnic groups; for example, p.F709del/F710del is the predominant mutation in Japanese patients [11,12]. Almost all of these mutations result in the total absence of functional enzyme and complete disruption of glycogen breakdown in muscle; however, in very rare cases a mild phenotype with minimal residual myophosphorylase activity (1%–2.5% of normal) occurs [13]. Patients with MD typically have childhood onset of exercise intolerance with symptoms including premature fatigue, myalgia, and/or muscle cramps [14,15,16]. Basal serum creatine kinase (CK) activity is elevated in MD patients, which indicates skeletal muscle damage. Older patients occasionally have muscle weakness and wasting [17].

## 2. Compensatory Energy Transfer Pathways

### 2.1. Adenine Nucleotide Degradation

**Figure 1:**
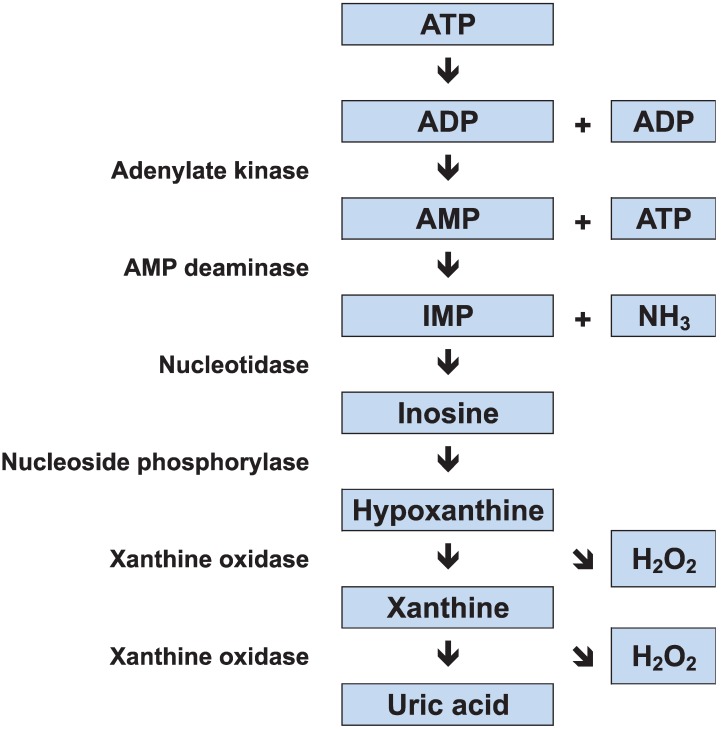
Adenine nucleotide degradation. ATP, adenosine triphosphate; ADP, adenosine diphosphate; IMP, inosine monophosphate; NH3, ammonia.

Skeletal muscles use three major metabolic processes to produce adenosine triphosphate (ATP): (1) oxidative phosphorylation; (2) glycolysis; and (3) adenylate kinase and creatine kinase (CK) reactions. The store of ATP in skeletal muscle is limited, and it would be used up in a few seconds of sprinting if not replenished; therefore the rate of ATP resynthesis must closely match the rate of consumption. More energy is available from oxidative phosphorylation (aerobic ATP production) than from glycolysis and adenylate kinase/CK reactions, but these anaerobic processes can be activated more rapidly than oxidative phosphorylation. In the adenylate kinase pathway, two adenosine diphosphate (ADP) molecules combine to regenerate ATP, and adenosine monophosphate (AMP) is produced as a by-product. This reaction is coupled with AMP deamination, resulting in the production of inosine monophosphate (IMP) and ammonia (NH_3_). IMP is metabolized to inosine and then to hypoxanthine, xanthine, and uric acid via xanthine oxidase (Figure 1).

During exercise, patients with MD have severely limited ATP resynthesis owing to both the absence of glycogenolysis and limited mitochondrial oxidative phosphorylation because of reduced substrate availability. Thus, the exercise intolerance in MD patients is caused by an imbalance between muscle energy demand and supply. The forearm exercise test has demonstrated that plasma lactate concentrations are not elevated in MD patients [1,16], which indicates increased alternative anaerobic pathway flux. Reports have shown abnormally large increases in muscle ADP [18], and plasma NH_3_ and hypoxanthine [19,20], in exercising MD patients; this suggests an increased adenine nucleotide degradation. This “emergency” mechanism for energy generation is blunted in healthy subjects. It has been shown that reliance on adenine nucleotide degradation during exercise leads to myogenic hyperuricemia in MD patients [21,22]. In addition, the reduction of hypoxanthine and xanthine to uric acid, which is catalyzed by xanthine oxidase, generates reactive oxygen species as a by-product; we recently found that MD patients experience elevated levels of oxidative stress [23].

### 2.2. Carbohydrate Metabolism

The breakdown of muscle glycogen is catalyzed by the enzyme myophosphorylase, which hydrolyzes α-1,4 glycosidic units to yield glucose 1-phosphate. The absence of this enzyme makes MD patients depend heavily on blood-borne fuels during exercise. Thus, it is well known that MD patients can perform prolonged exercise as a result of the “second wind phenomenon” that occurs owing to the improved delivery of extra-muscular fuels during exercise; intravenous glucose [24] or sucrose [25] administration also dramatically improves exercise tolerance in MD patients. Thus, the skeletal muscles of MD patients are able to take up glucose from the bloodstream via the glucose transporter GLUT4. Glucose is converted to glucose 6-phosphate and subsequently to pyruvate through glycolysis. It has been reported that MD patients take up more glucose during exercise than control subjects [24,26], possibly because of elevated GLUT4 protein content [27]. This improved glycolytic flux is very important when the availability of glycolytic metabolites is limited. 

A recent study demonstrated that the protein content of the monocarboxylate transporter MCT1, which facilitates the uptake of lactate, was higher in the skeletal muscles of MD patients than in healthy controls [28]. Lactate was once thought of as a metabolic waste product, but it is now known to be an oxidizable substrate after its conversion to pyruvate [29,30]. Moreover, lactate is oxidized more rapidly than other carbohydrates (fructose and glucose) during exercise in healthy subjects [31]. Therefore, lactate uptake via MCT1 could be an important mechanism by which MD patients increase the availability of pyruvate in their skeletal muscle.

### 2.3. Fat Metabolism

Enhanced fat oxidation may also help to compensate for the impaired muscle glycogenolysis in MD patients. It has been reported that the activity of β-hydroxyacyl CoA dehydrogenase—the key enzyme in the β-oxidation of fatty acids—was elevated in MD patients [32]. Likewise, a more recent study showed that fat mobilization and oxidation were higher in MD patients than healthy subjects [33]. Importantly, the same group also reported that the increased availability of free fatty acids during exercise did not augment fat oxidation in MD patients [34]. This is likely caused by the limitation in the flux of tricarboxylic acid cycle intermediates due to the limited intra-muscular pyruvate availability; in their words, “fat burns in the flame of carbohydrate” [33]. Thus, fatty acid availability alone does not determine the capacity for fat oxidation. It should be noted that another possible factor that may limit fatty acid oxidation is the transport of fatty acids into muscle and/or mitochondrial membranes via plasma membrane-associated fatty acid binding protein (FABPpm), fatty acid transport protein (FATP), and fatty acid translocase (FAT/CD36) [35,36]. However, at this time the expression of these fatty acid transporters has not been measured in MD patients.

### 2.4. Creatine-Phosphocreatine Shuttle

**Figure 2:**
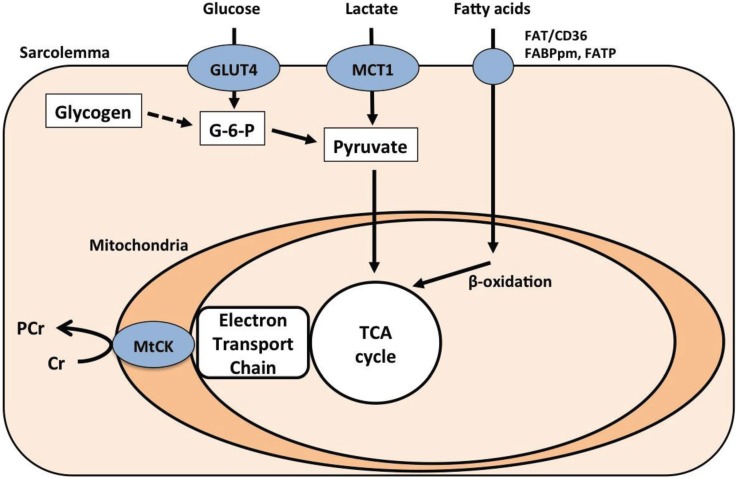
Schematic of metabolic pathways in the skeletal muscle of patients with McArdle disease. The dotted line indicates impaired glycogenolysis. Previous studies by the Tarnopolsky group have demonstrated increased GLUT4, MCT1, and MtCK protein levels in McArdle disease (MD) patients [27,28] FABPm, plasma membrane-associated fatty acid binding protein; FATP, fatty acid transport protein; G-6-P, glucose 1-phosphate; MCT, monocarboxylate transporter; mtCK, mitochondrial creatine kinase

The ATP yield from oxidative phosphorylation is transferred from the mitochondria to the cytosol by CK through the transfer of phosphate from phosphocreatine to creatine [37]. A recent study found specific up-regulation of mitochondrial CK (mt-CK) protein in MD patients without changes in total mitochondrial volume [28]. The enhancement of creatine-phosphocreatine flux by increased mt-CK is important for maintaining the phosphocreatine concentration and thus local ATP availability under the condition of reduced energy availability [38]. A previous study has shown that creatine supplementation improves skeletal muscle function in five of the nine MD patients [39]. However, the same group also reported that high dose creatine worsened the clinical features of exercise intolerance in patients with MD [40]. The authors speculated that this was due to insufficient physiological adaptation to the improvement in muscular electromechanical efficiency that occurs with creatine supplementation, leading to muscle hypercontractility during exercise and consequent worsening of the clinical symptoms of MD. Patients with MD compensate for their limited oxidative capacity not only by extra-muscular fuel uptake via metabolite transporters, but also through intra-muscular energy transfer between mitochondria and the site of energy consumption (Figure 2).

## 3. Exercise Training as a Possible Treatment

Since exercise triggers the clinical symptoms of MD, affected patients tend to avoid exercise and live sedentary lifestyles. Paradoxically, however, recent evidence has shown that carefully supervised exercise may help to reduce symptoms of exercise intolerance in MD [41,42]. For example, 14 weeks of moderate aerobic exercise training (cycling for 30–40 minutes at an intensity corresponding to 60%–70% of maximal heart rate, 4 times per week) increased the peak cardiac output of MD patients by 15% and the activity of mitochondrial enzymes, citrate synthase and hydroxyacyl CoA dehydrogenase, by 60%–80% [43]. In healthy subjects, it is well known that exercise training increases the expression of glucose [44,45], lactate [46,47], and fatty acid transporters [48,49] in muscle; in contrast, physical inactivity decreases the expression of these substrate transporters [50,51,52]. Although the effects of exercise training on metabolite transporters in MD patients have not yet been investigated, these data suggest that the capacity for delivery and oxidation of blood-borne fuels increases with exercise training in MD patients. Another study reported that MD patients who performed 8 months of low- to moderate-intensity aerobic exercise training (walking and/or cycling for 10–60 minutes at an intensity corresponding to 60% of maximal heart rate, 5 times per week) increased their peak power output by 25% and their peak oxygen uptake (VO_2peak_) by 44% [53]. Serum CK levels were also decreased after the training intervention, which likely indicates that less muscle damage was occurring as an adaption to the exercise program. Moreover, surprisingly, it has been reported that a 38-year-old male patient with MD was able to run 10 km in 60 minutes after 4 months of training; his VO_2peak_ increased from 14.6 to 30.8 mL/kg/min over the training period [54]. Finally, resistance (weight lifting) exercise may also have positive effects for MD patients; a recent report showed that 6 weeks of resistance training at 60%–75% of the one-repetition-maximum (2 sessions per week) resulted in a 27% increase in bench press performance in a 15-year-old male patient with MD [55]. Collectively, exercise training with pre-exercise carbohydrate ingestion may benefit MD patients, whereas isometric exercise should be discouraged.

## 4. Conclusions

Previous studies have revealed some novel energy transfer mechanisms that partially compensate for the absence of glycogenolysis in patients with MD. Since MD patients rely on blood-borne fuels during exercise, their capacity for extra-muscular fuel uptake via specific substrate transporters is elevated. This ultimately results in increased pyruvate availability within the muscle. Consequently, pre-exercise carbohydrate ingestion is at present considered the most beneficial intervention for MD patients. In combination with this nutritional intervention, carefully supervised exercise training may benefit MD patients. Finally, knock-in mice for the R50X mutation in the PYGM gene have been developed [56]. This genetically modified animal model of MD will hopefully reveal new therapeutic approaches for this disease in future studies.
